# The Insulin and IGF-I Pathway in Endocrine Glands Carcinogenesis

**DOI:** 10.1155/2012/635614

**Published:** 2012-08-08

**Authors:** Roberta Malaguarnera, Alaide Morcavallo, Antonino Belfiore

**Affiliations:** Endocrinology, Department of Health Sciences, Magna Græcia University of Catanzaro, Campus Universitario, Località Germaneto, 88100 Catanzaro, Italy

## Abstract

Endocrine cancers are a heterogeneous group of diseases that may arise from endocrine cells in any gland of the endocrine system. These malignancies may show an aggressive behavior and resistance to the common anticancer therapies. The etiopathogenesis of these tumors remains mostly unknown. The normal embryological development and differentiation of several endocrine glands are regulated by specific pituitary tropins, which, in adult life, control the function and trophism of the endocrine gland. Pituitary tropins act in concert with peptide growth factors, including the insulin-like growth factors (IGFs), which are considered key regulators of cell growth, proliferation, and apoptosis. While pituitary TSH is regarded as tumor-promoting factor for metastatic thyroid cancer, the role of other pituitary hormones in endocrine cancers is uncertain. However, multiple molecular abnormalities of the IGF system frequently occur in endocrine cancers and may have a role in tumorigenesis as well as in tumor progression and resistance to therapies. Herein, we will review studies indicating a role of IGF system dysregulation in endocrine cancers and will discuss the possible implications of these findings for tumor prevention and treatment, with a major focus on cancers from the thyroid, adrenal, and ovary, which are the most extensively studied.

## 1. Introduction

Endocrine malignancies, including carcinomas of the thyroid, adrenal, and ovary, are relatively rare tumors deriving from cells present in endocrine glands. Surgery is currently the treatment of choice for these tumors and is often successful in early stages of disease. However, this therapeutic approach for the advanced tumors remains unsatisfactory and is associated with poor prognosis. Thus, a better understanding of the molecular mechanisms and the critical intracellular networks underlying endocrine oncogenesis may help in discovering new targets that could represent promising therapeutic options for these malignancies. As specific pituitary tropins control the trophism and function of specific endocrine glands, it is tempting to speculate about a possible role for these pituitary hormones in endocrine glands tumorigenesis. However, this assumption is controversial as other signaling effectors, including the IGF system, have often a major role in endocrine tumorigenesis.

This paper covers the recent molecular advances in this field focusing on the role of the IGF system in endocrine tumorigenesis with particular attention on the endocrine cancers best characterized until now (i.e., thyroid, adrenal, and ovarian tumors).

## 2. Regulation of Thyroid, Adrenocortical, and Ovarian Tumor Growth: The Role of Pituitary Hormones

Thyroid cancer growth regulation has been extensively characterized. Several molecular alterations associated with thyroid tumorigenesis have been identified and often converge into the activation of MAPK (mitogen-activated protein kinase) and PI3K (phosphatidylinositol-3-kinase) signaling pathways [1].

Thyroid gland function and trophism is mainly regulated by thyrotropin hormone (TSH). TSH is considered the key player of thyrocyte differentiation and proliferation. Its mitogenic actions are mainly mediated by cAMP, which in turn activates protein kinase A (PKA) dependent and independent pathways. Activating mutations of the TSH receptor (TSHR) or of the gene encoding the G_s_ *α* subunit of the heterotrimeric G protein that couples TSHR to adenylyl cyclase (GSP) have been described in 30% of autonomously functioning thyroid adenomas while they are rare in thyroid carcinomas [2, 3]. TSH, however, has a well-known promoting role for thyroid cancer metastases, and TSH suppressive therapy with L-thyroxine is a well-established therapy in the postoperative management of differentiated thyroid cancer [4]. To exert its maximal mitogenic effects, TSH requires concomitant ligand-activated tyrosine kinase receptor (RTK) signaling. Studies carried out in thyroid cell cultures have especially highlighted the importance of the IGF system in regulating thyroid cell growth in response to TSH [5, 6].

TSH makes the cells competent to progress into the G1 phase in response to insulin or IGF-I, which can thus be qualified as the only genuine mitogens [7]. In fact, the protumorigenic effects of TSH are irrelevant in the absence of growth factors, but they are greatly potentiated by the presence of insulin or IGF-I at physiological concentrations [5, 8]. Furthermore, we have recently reported a key role of the IGF system in the biology of follicular thyroid progenitor/stem cells [9]. Insulin/IGF-I signaling pathways are important also in the regulation of thyroid-specific genes transcription, including the TSH receptor [10], thyroglobulin (Tg), and thyroperoxidase (TPO) [11, 12]. Gene expression of both Tg [13] and TPO [14] is mediated predominantly by thyroid transcription factor-2 (TTF-2), a thyroid-specific transcription factor that binds to the promoter of both genes [15, 16] and is stimulated by both the cAMP and the insulin/IGF-I pathways, which may have additive effects [17].

Regarding the adrenocortical cancers, their molecular pathogenesis is still incompletely understood. In contrast to thyroid carcinomas, the cAMP/PKA pathway seems to be less involved in the development of these tumors. Although pituitary adrenocorticotropic hormone (ACTH) stimulates adrenal function by inducing steroidogenic enzymes and increases adrenal gland weight, the proliferative action of ACTH for adrenal tumors has been questioned, and opposite effects, under defined cell culture conditions, have been reported. *In vitro* inhibition of adrenal cell proliferation by physiological ACTH concentrations has been reported by several groups [18–21]. In support of the growth-inhibiting effect of ACTH, no activating mutations of the ACTH receptor have been found in benign or malignant adrenocortical tumors [22, 23]. Conversely, allelic loss of the ACTH receptor gene has been reported in a subset of sporadic benign and malignant adrenocortical tumors where it was associated with undifferentiated phenotype and worse prognosis [24]. These data tend to exclude a role of ACTH receptor as putative oncogene in adrenal oncogenesis while supporting its role as tumor suppressor. In summary, in the adrenal cortex, the ACTH/PKA signaling is mainly involved in regulating steroid hormone synthesis and cellular differentiation rather than in controlling cellular proliferation and tumor growth. Similarly to thyroid cancer, molecular alterations frequently observed in adrenocortical carcinoma include deregulation of the IGF system as well as mutations in p53 and RAS [25]. In addition to IGF-II overexpression, increased levels of the IGF-IR and IGFBP-2 have been found in advanced human adrenal carcinomas, resulting in increased IGF-dependent cell proliferation and inhibition of the ACTH antiproliferative effect. Although the functional significance of the strong and specific overexpression of IGFs in adrenocortical carcinomas remains still unknown, these factors may regulate both steroidogenic and mitogenic effects and, similarly to what is seen in thyroid cancer, establish autocrine positive loops that promote growth advantage and transformation toward a more malignant phenotype.

In ovarian cancers, the role of the pituitary tropins is still controversial. Pituitary LH and FSH lead to increased sex steroids secretion which may favor ovarian cancer development [26, 27]. A role for gonadotropins in ovarian tumorigenesis is also supported by the observation that ovarian cancer incidence reaches a peak in the postmenopausal period, during which FSH levels are particularly high [28]. Yet, a study on normal rabbit ovarian surface epithelium showed that FSH and LH/hCG stimulate growth *in vitro* [29]. However, controversial results have been obtained by different research groups. For instance, a more recent study has reported no increase in cell proliferation with LH [30]. Although the mechanisms by which FSH and LH stimulate or inhibit the proliferation of ovarian epithelium remain still unknown, these hormones exert their effects interacting with their specific receptors. Some ovarian cancers, especially those poorly differentiated, lose FSH receptor (FSHR) expression [30]. This observation suggests that FSH may be a growth-promoting factor important at early stages of ovarian epithelial tumorigenesis, with some ovarian tumors losing their requirement for FSH later in tumor development. Like adrenal and thyroid cancers, also in ovarian tumors, IGF system components are often overexpressed. IGF-II appears to increase proliferation and induce differentiation in granulosa cells via the IGF-IR and synergizes with FSH to induce steroidogenesis as well as mitogenesis [31]. This synergism likely involves IGF-induced upregulation of the FSHR [31, 32] and/or the involvement of intracellular mechanisms leading to the activation of intracellular pathways (i.e., protein kinase C, cAMP, MAPK, and PI3K).

**Figure 1:**
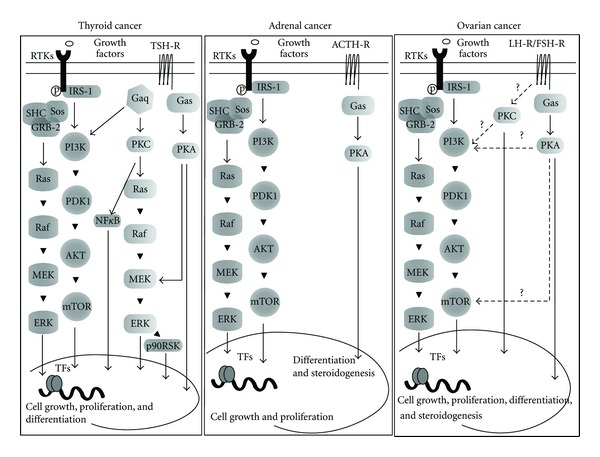
Schematic representation of the interplay between pituitary hormones and main signaling pathways of the IGF system in thyroid, adrenal and ovary cancers. Dot lines: proposed signaling pathways.

A schematic representation of the interplay between pituitary hormones and main signaling pathways of the IGF system in thyroid, adrenal, and ovary cancers is shown in Figure 1.

Altogether, the lines of evidence reported in these three tumor histotypes suggest that, although the specific pituitary tropin exerts an important role in regulating the growth, differentiation, and function of the target endocrine gland, the interplay of pituitary hormones with other factors, such as the IGF system, is crucial for deregulated cell proliferation and transformation. The IGF system may represent, therefore, a promising therapeutical target for these tumors.

## 3. The IGF System and Its Involvement in Cancer

In mammals the IGF system includes four receptors (the insulin receptor (IR), the IGF-I receptor (IGF-IR), the insulin-receptor-related receptor (IRR), and the Mannose-6-phosphate/IGF-II receptor (IGF-IIR)), four ligands (proinsulin, insulin, IGF-I, and IGF-II), and six high-affinity binding proteins (IGFP-1 to 6). The human IR exists in two isoforms (IR-A and IR-B) generated by alternative splicing of the IR gene with the exclusion (IR-A) or inclusion (IR-B) of 12 amino acids encoded by exon 11. The IR and the IGF-IR have highly homologous structure, but different functions. Given the high degree of homology, IR and IGF-IR can heterodimerize leading to the formation of insulin/IGF-I hybrid receptors (HRs) [33, 34]. The IGF-IIR is a structurally distinct cell surface receptor whose major function is to induce internalization and degradation of IGF-II, thus modulating its extracellular levels [34].

 With regard to the ligands, insulin and IGFs are related peptides, involved in metabolism as well as in growth and reproduction. Insulin largely circulates in free form while more than 90% of IGFs circulates bound to a complex family of IGF-binding proteins (IGFBPs), which regulate both the half-life and the biological effects of IGFs [35]. Insulin and IGFs bind with different affinity IR isoforms and IGF-IR (for more details see [36–38]).

Recently, it has been reported that proinsulin, the insulin prohormone, which is characterized by low metabolic activity compared to mature insulin, is a selective IR-A ligand and may exert a putative role on growth and cell proliferation [39, 40].

After ligand binding, phosphorylated receptors activate two main signaling pathways, the PI3K and the MAPK cascade, involved in the regulation of cell metabolism, proliferation, and survival. Although both the IR and IGF-IR similarly activate these signaling networks, subtle differences exist in the recruitment of certain intracellular mediators and substrates between the two receptors, leading to the specific biological effects of each hormone. Details regarding the IGF system have been previously covered by several reviews to which we refer for more information [34, 36, 41].

Since the IGF system exerts a pivotal role in cell growth and homeostasis, it is not surprising that aberrant expression of receptors belonging to this system might be involved in cancer development, progression, and metastasis. The key role of IGF-IR in oncogenic transformation derives from the studies showing that IGF-IR null cells cannot be transformed by several cellular or viral oncogenes, whereas they become susceptible to the oncogenic mediated transformation after the reintroduction of a functional IGF-IR [42, 43]. However, increased levels of IGF-IR do not result in autonomous receptor signaling in the absence of IGF ligand, while it can induce malignant transformation in presence of its specific ligands [44]. Similarly, in estrogen responsive breast cancer cell lines, growth response to insulin could be specifically inhibited by anti-IR but not anti-IGF-IR blocking antibodies while it can be mimicked by an anti-IR stimulating antibody [45]. These data are in agreement with studies indicating that IR-transfected cells acquire insulin-dependent malignant changes [46, 47] and support the notion that IR may elicit mitogenic and antiapoptotic effects similar to IGF-IR, contributing to cancer development and progression. The first direct evidence that IR may be overexpressed in cancer cells was reported by Papa et al. in breast tumors [37]. Subsequent studies demonstrated that IR is also overexpressed in other human malignancies, including endocrine tumors, such as cancer of the thyroid, ovary, and adrenal glands [38, 48–50]. In most of these tumors, cell growth is dependent on IR activation by insulin, suggesting a mitogenic role of this hormone [51], although IR, isoform A, may also be activated by IGF-II [46, 52, 53]. Both IR isoforms may be overexpressed in cancer, but usually IR-A is predominant, representing 60–100% of total IR. Data showing an increased relative abundance of IR-A are also available for certain endocrine cancers [38, 48, 54]. This observation is particularly interesting, as IR-A is mainly expressed in fetal life, while IR-B predominates in differentiated tissues [38, 55]. Furthermore, at variance with IR-B, which is a highly specific receptor for insulin, IR-A is a high-affinity receptor for insulin; it shows intermediate affinity for IGF-II and low affinity for IGF-I [38]. Although IGF-II is able to bind both to IGF-IR and IR-A with similar affinity, its binding to IR-A has important implications. Indeed, IR-A overexpression amplifies IGF-II effects in cancer cells and serves as a signaling diversification factor, as IR-A and IGF-IR activate different downstream signals.

Because of the high homology between the IR and the IGF-IR [56], in cells coexpressing IRs and IGF-IR [57] hybrid IR/IGF-IR receptors (HRs) may form [58–60]. Functionally, HRs are considered high-affinity IGF-I-binding sites as they bind insulin with much lower affinity [60]. In thyroid cancers large amounts of HRs have been measured both in well-differentiated papillary carcinomas and in poorly differentiated/undifferentiated carcinomas, probably as a consequence of increased IR expression [33]. In these tumors, HRs account for 50–75% of the total IGF-I binding sites and mediate IGF-I mitogenic signaling. No data are available regarding HRs expression in other endocrine malignancies.

### 3.1. IR and IGF-IR Signaling Pathways: Relevance to Endocrine Malignancies

IR and IGF-IR share many similarities not only in their structures but also in their downstream signaling pathways. Upon ligand binding, the intrinsic tyrosine kinases of both IR and IGF-IR are activated and this results in the phosphorylation of several receptor substrates including the components of the IRS family and Shc. These substrates, in turn, act as multisite “docking” proteins for kinases and adaptors, such as PI3K, Syp, Fyn, Nck, and Grb2, which trigger the activation of downstream kinase cascades [61]. IRS proteins are also involved in the crosstalk with other signaling pathways, including those coming from other growth factors [62], cytokines [63], and integrins [64].

The two main signaling pathways downstream to IR and IGF-IR include the mitogen-activated protein kinases cascade (MAPKs), which involves the sequential activation of a cascade of serine/threonine protein kinases with a key role in the regulation of cellular proliferation and gene expression and the PI3K signaling pathway, which mediates metabolic actions but also stimulates cell growth and survival. Both MAPK and PI3K pathways enhance protein synthesis through mTOR activation and trigger antiapoptotic effects through the phosphorylation and inactivation of Bad [65]. Molecular alterations (mutational and nonmutational) in both PI3K and MAPK have been reported in several malignancies including those from thyroid, ovary, and adrenal glands.

Conditional or constitutive deregulation of MAPK and PI3K cascades is a common event in thyroid cancer and may play a pathogenetic role in this tumor [1]. Indeed, deregulated activation of the MAPK cascade via mutations and/or rearrangements in RET, RAS, and BRAF genes occurs in ~70% of papillary thyroid carcinomas (the most common subtype of thyroid cancers) [66–68]. Thyroid carcinomas also show mutations in PI3K signaling effectors such as PTEN and phosphoinositide-3-kinase, catalytic, alpha polypeptide (PIK3CA). PTEN is downregulated in ~37% of well-differentiated thyroid carcinomas and downregulated or lost in >50% of highly malignant thyroid cancers [69]; point mutations or copy number changes in PIK3CA are found in ~23% of anaplastic thyroid cancers where they can coexist with either RAS or BRAF mutations [70].

In adrenal cancer, pathway analysis for the genomic regions associated with poor prognosis has shown deletions of genes that negatively regulate the activation of ERK1/2 and loss of PTEN gene [71]. Yet, several reports have identified activating RAS mutations [72, 73], while only two papers have analyzed mutations in BRAF gene and found that their prevalence is low [74, 75]. Although functional studies are needed to better characterize the effect of these mutations in adrenocortical tumors, it is possible that deregulation in the MAPK pathway may significantly contribute to aggressive phenotypes.

In ovarian cancer, mutually exclusive mutations of KRAS and BRAF have been described in about 30–50% of low-grade tumors [76–79], while they are rare in high-grade tumors. RAS mutations may promote ovarian tumorigenesis not only through MAPK but also via the interaction with the PI3K/AKT pathway. In ovarian cancers PI3K activation, occurring via either PIK3CA gene amplification/mutations or PTEN protein loss, has been reported by several studies [80–83] with the highest frequency in most malignant histotypes [84].

In the context of the three endocrine tumors mentioned above, the dysregulation of the IGF system may represent one nonmutational mechanism activating MAPK and PI3K signaling cascades. The increased IGF-IR-mediated activation of MAPK/PI3K signaling may, in turn, induce IGF-IR and/or its ligands expression and reduce the expression of IGFBP-3 [85–87]. This close relationship between the IGF system and MAPK/PI3K-mediated signals may contribute to cancer development, progression, invasion, and aggressive behavior.

Indeed, via PI3K/AKT/PTEN and ERK dependent mechanisms [88–90], IGFs control several cycle checkpoints, in particular the G0-G1 transition, increasing cyclin D1 and CDK4 gene expression and down-regulating the cyclin-dependent kinase inhibitor (CDKI) p27. Moreover, through the same pathways, IGFs regulate cell invasion and tumor-dependent angiogenesis modulating the expression of molecular mediators of extracellular matrix remodeling and degradation including type IV collagenases, matrix metalloproteinase-2 (MMP-2) and matrix metalloproteinase-9 (MMP-9), and the membrane type 1 MMP (MMP-14) [91, 92]. These enzymes play an important role in malignant progression and metastatic spread of solid tumors, including endocrine ones. MMPs expression has been found to be elevated in papillary thyroid cancer as compared with normal thyroid tissue [93, 94]. A strong MMP2 expression has been also found in malignant adrenal tumors and considered an unfavorable prognostic factor [95]. In ovarian cancers, MMPs are frequently overexpressed and appear to be an early event of ovarian tumorigenesis suggesting a role of these enzymes in ovarian tumor initiation and not only in tumor progression and invasion [96].

Relevant crosstalks between the IGF system and other signaling pathways also include the involvement of the janus kinase (JAK)-1/2 mediated signaling and the activation of transcription proteins STAT. In particular, STAT-3 may be required for the maintenance of transforming activity of IGF-IR [97]. IGF-I is able to activate STAT-3, but not STAT-5, and this activation is probably mediated by JAK proteins [98]. These mechanisms have been demonstrated in several models, and they may also occur in endocrine cancers, where both STAT and IGF signaling play an important role in tumor invasion and metastasization. Indeed, the STAT-3 pathway is significantly upregulated in metastatic thyroid papillary cancers, suggesting a potential role for activated STAT-3 in lymphatic metastases [91]. Yet, in both ovarian and adrenal cancers a role for STAT signaling in invasion and cancer prognosis has been also identified [99, 100].

Other molecules interacting with the IGF system and involved in the pathogenesis of thyroid, adrenal, and ovarian cancers include the tumor suppressor p53. Inactivating mutations of p53 gene occur in 10%, 47%, and 25% of thyroid, ovary, and adrenal sporadic carcinomas [101, 102], respectively. However, in all these tumors, also when not mutated, p53 activity may be inhibited by other mechanisms among which are an unbalanced expression of isoforms with a dominant negative function, the interaction with Mdm2, and the cooperation with other members of the p53 family such as TAp63*α*, TAp73*α*, and their dominant negative variants (ΔNp63 and ΔNp73) [103]. The activity of wild-type p53 reduces IGF axis activity by multiple mechanisms which include inhibition of IGF-IR [104], IR [105], and IGF-II expression [106] with a concomitant increase of IGFBP-3 transcription [107]. Therefore, aberrant p53 (i.e., p53 lacking its suppressor function through point mutations or via other mechanisms) greatly enhances the activity of IGF axis at multiple levels [104]. In the three endocrine tumors mentioned above, the crosstalk between the IGF system and p53 appears an important prerequisite for oncogene-driven tumor cell trasformation, cancer progression, and resistance to anticancer therapies.

### 3.2. Circulating Levels of Insulin and IGFs and Endocrine Cancers

Epidemiological studies have shown that elevated plasma concentrations of IGFs are associated to increased risk for the development of several human malignancies including cancers of the breast, colon, and prostate as well as sarcomas [61, 108–113]. For instance, several studies have provided strong evidence that premenopausal, but not postmenopausal, women in the highest tertile of serum IGF-I levels had an increased risk of developing breast cancer [114], and that a high IGF-I : IGFBP-3 ratio may be associated with greater breast density and increased breast cancer risk [108, 115].

High circulating IGFs concentrations may exert biological effects in malignant cells not only through IGF-IR but also via IR-A and HRs. As described in more detail below, all these receptors are overexpressed in endocrine cancers [31, 48] as well as in thyroid cancer stem-like cells [9].

A possible role for serum IGF-I in thyroid cancerogenesis has been suggested by the observation that acromegalic patients, who are exposed to sustained high serum IGF-I levels, show an increased frequency of thyroid cancer [116, 117]. In adrenocortical tumors high serum IGFs and low IGFBP3 levels are correlated with cancer risk and are predictive of metastases development [111, 118].

Finally, some haplotypes and SNPs in the IGF components may influence ovarian risk, either directly or by increasing the IGF-I plasma levels. In particular, the following SNPs in the IGFBPs (rs10228265, rs4988515, rs2270628, rs2854746, and rs2854744), in IGF-I (rs11111285, rs1996656 and rs1019731), and in IGF-II (rs4320932, rs4244809, rs680, rs1003483, and rs7924316) have been associated with increased ovarian cancer risk [119, 120].

Not only IGFs but also circulating insulin has been suggested to be involved in the tumorigenesis process. Indeed, a number of population studies have provided substantial and circumstantial lines of evidence that insulin resistance and hyperinsulinemia, common factors underlying obesity and type 2 diabetes mellitus (T2DM), are strong candidates for the increased cancer risk associated with these disorders [121–123]. Although insulin is considered a hormone regulating energy metabolism, it also exerts proliferative, antiapoptotic, and migratory actions, collectively indicated as “mitogenic effects,” via its own receptor (IR). This observation is known from long time and helps in understanding the link between insulin resistance/hyperinsulinemia and cancer.

The involvement of insulin in cell trasformation and cancer development was firstly suggested by *in vivo* evidence that administration of insulin induced growth of mammary tumor in mice [124] and promoted aberrant crypt foci in the colon of rats [125–127], while insulin deficiency or calorie restriction exerted a protective role [124]. Similarly, in obese mice, insulin levels were positively associated with the proliferation of transplanted lung and colon cancer cells [127].

In light of these experimental lines of evidence, clinical studies have been conducted to investigate the possible role of hyperinsulinemia and insulin resistance in endocrine tumors. At this regard, several case-control and prospective studies have found a strong positive association between overweight/obesity and thyroid cancer risk [128–137], although the data are not entirely consistent [138–145].

The exact nature of the relationship between body mass index (BMI) and thyroid cancer incidence remains still unclear. Besides the high circulating levels of insulin present in overweight/obese patients, other potential mechanisms may include increased levels of inflammatory adipokines [146]. In addition, although obesity is associated with poor prognosis for several malignancies, this relationship has not been reported for thyroid cancer [142].

In partial support with the finding that hyperinsulinemia and insulin resistance are risk factors for thyroid cancer, studies conducted in T2DM patients have shown that higher fasting glucose levels are associated with increased thyroid cancer risk [144, 147]. However, also for this association conflicting results have been obtained [134].

Concerning the association between hyperinsulinemia/insulin resistance and ovarian cancer risk, several lines of evidence suggest that women affected by polycystic ovary syndrome (PCOS), a condition associated with insulin-resistance, are more likely to develop ovarian cancer (OR, 2.5; 95% CI 1.08–5.89) [148]. Furthermore, a meta-analysis of ten cohort studies has shown that overweight and obesity are associated with higher ovarian cancer mortality (OR, 1.6; 95% CI 1.1–2.34) and that, among patients with advanced ovarian cancer, premorbid obesity is associated with worse prognosis (OR, 1.5; 95% CI 1.09–1.93) [149]. However, other studies have not supported these results [150], suggesting that further investigation is needed to firmly establish the association between ovarian cancer and insulin resistance [151].

Finally, it has been suggested that adrenal incidentalomas, usually benign tumors, might be related to hyperinsulinemia and insulin resistance. This hypothesis was postulated for the first time by Reincke et al. [152], who observed a proliferative effect of insulin on adrenal cancer cells without effect on cortisol synthesis [152]. However, a causative role of adrenal incidentalomas for metabolic syndrome cannot be excluded, as some patients show a slight hypercortisolism that may contribute to the insulin resistance. In fact, surgical tumor resection may revert or ameliorate these metabolic alterations [153]. Regarding the link between hyperinsulinemia and malignant adrenal tumors, scanty data are present in the literature so far.

## 4. IGF System Abnormalities in Specific Endocrine Cancer and Possible Therapeutical Implications

### 4.1. Thyroid Cancer

Human thyroid carcinomas derived from the thyroid follicular cells (TFCs) include a variety of histotypes ranging from well-differentiated (papillary and follicular) to undifferentiated (anaplastic) cancers. Altogether, they represent approximately 1% (3% in women) of all human cancers [154, 155].

Well-differentiated thyroid carcinomas account for approximately 90% of all thyroid cancers. They retain a variable degree of TSH responsiveness and have a mortality rate of approximately 10%. Poorly differentiated and undifferentiated carcinomas account for only approximately 10% of all thyroid cancers; they have weak or no TSH responsiveness and have a mortality rate ranging from 50% to 100% [48, 156].

As previously mentioned, the IGF-I system plays an important role in regulating normal growth and development in the thyroid [6, 9] and appears also to be involved in thyroid tumorigenesis.

The coexpression of IGF-I and its cognate receptor, IGF-IR, has been documented by various studies in both cultured thyroid cells and tissue specimens. In particular, cultured human and ovine thyrocytes are able to release IGF-I in the culture media [157, 158]. Also, thyroid adenoma cell lines synthesize IGF-I, which stimulates cell growth by autocrine mechanisms [159]. Immunoreactive IGF-I and IGFBPs were also found in the extracts of normal and nodular thyroid tissue specimens obtained at surgery from patients with nontoxic goiter [160–162] (Table 1).

Functional IGF-IR is usually expressed at high levels in thyroid cancer cells. In SW579 thyroid carcinoma cells IGF-I induced angiogenic activity via increased synthesis of HIF-1*α*  transcription factor and consequent stimulation of vascular endothelial growth factor (VEGF) expression [163]. Belfiore et al. measured IGFs and cognate receptors in both thyroid cell lines and tissue specimens. IGF-I content ranged from 104 to 2566 nM/g in cancer tissue and 69 to 680 nM/g in normal thyroid tissue. By using a specific ELISA, they also found that IGF-IR is overexpressed in both thyroid cancer cell lines and specimens as compared to the normal tissue [33] (Table 1).

IGF-IR overexpression in thyroid cancer specimens has been also found by using immunohistochemistry and in situ hybridization, and IGF-I was found to be produced in either paracrine or autocrine manner [8, 164]. IGF-I and IGF-IR immunoreactivity was found to be increased both in adenomas and carcinomas compared with normal thyroid. IGF-I overexpression was more marked in the undifferentiated and poorly differentiated histotypes of thyroid cancer [165].

The above-mentioned study of Vella et al. demonstrated that thyroid cancers overexpress not only IGF-I and IGF-IR, but also IGF-II and IR. In particular, the IGF-II/IR-A autocrine loop is especially activated in poorly differentiated and anaplastic cancers. The relative abundance of IR-A also increases in dedifferentiated cancers. In this context, the IGF-IR seems less important than IR-A in mediating IGF-II mitogenic effects, and blocking antibodies to IR markedly reduced the effects of IGF-II [48].

The concomitant high expression of both IGF-IR and IR-A in thyroid cancer cells causes overexpression of IR/IGF-IR hybrid receptors, which, in most cases, exceed the IGF-IR content. In cells with a high IR/IGF-IR content, blocking antibodies specific to these receptors substantially inhibited IGF-I-induced cell growth. These data indicate that, in addition to IGF-IR and IR-A, also IR/IGF-IR hybrids may be a target in thyroid cancers [33] (Table 1).

Progenitor/stem cells are increasingly considered to be at the origin of most malignancies [166]. Therefore, we recently isolated progenitor/stem cells from both normal and cancer specimens and cultured them as thyrospheres, in order to study the IGF system in this model [9]. We found that IGF-I and IGF-II are produced at high levels by all thyrospheres. However, the IGF-I : IGF-II ratio was approximately 5 : 1 in normal thyrospheres whereas it was 1 : 1 in cancer thyrospheres. IR and IGF-IR in human thyrospheres were markedly overexpressed and with a higher IR : IGF-IR ratio as compared to primary cultures. The IR : IGF-IR ratio was also higher in cancer than in normal thyrospheres. Receptors (IR and IGF-IR) and ligands (IGF-I and IGF-II), all expressed at high levels in thyrospheres, markedly decreased in differentiating cells. IR-A was the predominant isoform in thyrospheres, especially from cancer, while IR-B was predominant in differentiating cells. IR-A relative abundance was associated with characteristics of stemness and with cancer: it ranged from 65 to 86% in cancer thyrospheres, from 50 to 65% in normal thyrospheres, and from 40 to 45% in normal thyroid primary cultures or differentiated sphere-derived thyrocytes [9]. The expression of IR, IGF-IR, and their ligands was evaluated by quantitative real-time PCR. Western blot analysis for IR and IGF-IR confirmed PCR data. Cancer thyrosphere growth was stimulated by insulin and IGFs, while IGF-II was most potent in inducing cell renewal [9] (Table 1).

Considering the involvement of IGF-I system in thyroid cancer [9, 55, 156], Wang et al. studied the potential therapeutic role of anti-IGF-IR humanized monoclonal antibody A12 both *in vitro* and *in vivo*. In accordance to other studies, they found that IGF-IR is expressed in various human thyroid cancer cell lines and in normal and neoplastic human thyroid tissues, including surgical specimens of papillary and anaplastic carcinomas. IGF-IR antibody A12 was able to significantly inhibit the proliferation of cultured anaplastic cancer cells by downregulating the IGF-IR signaling pathway. Moreover, administration of A12 also reduced tumor volume in an orthotopic anaplastic cancer nude mouse model and prolonged survival [167] (Table 2).

The PPAR*γ* agonists thiazolidinediones and biguanides (metformin) are used as antidiabetic drugs for their insulin-sensitizing effect achieved by different mechanisms [168]. Because of these effects, both these classes of drugs lower circulating insulin levels, and, in principle, they may have favorable effects in patients with IR-overexpressing tumors. Moreover, both thiazolidinediones and metformin have direct and pleiotropic anti-IGF effects in cultured cancer cells. In particular, in anaplastic thyroid cancer (ATC) cells, rosiglitazone antagonized the biological effects of IGF-I by upregulating phosphatase and tensin homolog deleted from chromosome 10 (PTEN) and consequently inhibiting the phosphatidylinositol 3-kinase (PI3K)/Akt signaling pathway. As a consequence, it reduced anchorage-dependent and -independent growth and migration, increased apoptosis rate, and induced partial redifferentiation in these cancer cells [169]. Rosiglitazone also potentiated the antitumor effect of doxorubicin (Table 2).

Recently, Chen et al. evaluated the effects of metformin, in ATC cell lines and in thyroid cancer stem cells. They found that metformin antagonized the growth-stimulatory effect of insulin in thyroid cancer cell lines. Specifically, metformin inhibited cell cycle progression, inhibited clonal cell growth, and reduced thyroid cancer sphere formation. Moreover, the metformin potentiated the antimitogenic effect of chemotherapeutic agents, such as doxorubicin and cisplatin, in ATC cells [170] (Table 2).

### 4.2. Adrenal Gland Cancer

Adrenal tumors are classified into benign and malignant groups. Tumor histotypes can be either hormonally silent or hormone secreting. In this case tumors may produce glucocorticoids, androgens, mineralocorticoids, estrogens, and combinations thereof [171]. The vast majority of adrenocortical tumors are benign, while adrenocortical carcinomas (ACCs) are relatively rare; they presents with extremely poor prognosis as a consequence of metastases or local invasion [172]. The frequency of small benign adrenocortical tumors increases with age, ranging between 3 and 7% of all adrenal carcinomas in adults over 50 years. However, ACCs account for only 0.05–0.2% of all cancers [173], with an estimated incidence between 1 and 2 per million and per year in adults in North America and Europe [174, 175]. In children, the incidence is approximately 10-fold lower except in South Brazil where there is a high incidence of pediatric ACC [176].

As previously mentioned, the IGF system has a physiological role in normal adrenal growth and development [177] and is also involved in ACC proliferation and progression [178]. An increased expression of the IGF-IR was demonstrated in SW13 and in H295R human adrenocortical carcinoma cell lines [179]. Both IGF-I and IGF-II are produced by H295R cells [180], and IGF-II increases during proliferation. In the same cell model an IGF-IR blocking antibody (*α*-IR3) was able to hamper cell growth, demonstrating that autocrine IGF-II production may stimulate cell growth through the IGF-IR [181] (Table 1).

In the reticularis layer of normal adrenal tissues a large number of IGF-I-positive cells with granular cytoplasmic (GC) staining pattern are present [182]. The proportion of these cells increases with the tumorigenesis process; hyperplastic glands show 10–50% of IGF-I-positive cells, while adenomas and carcinomas have over 50% of IGF-I positive cells in 64% and 83% of cases, respectively. Similarly, the IGF-IR is more expressed in adenomatous adrenal tissues than in nontumoral tissues [179]. IGF-II is one of the most expressed genes in adrenocortical carcinomas [183, 184]. The IGF-II gene is located at locus 11p15, which is maternally imprinted and consequently expressed only from the paternal allele. Structural abnormalities, characterized by the loss of maternal allele with the duplication of paternal allele, lead to biallelic expression of IGF-II gene. These alterations are frequently observed in sporadic adult ACCs, but only rarely in adenomas [118, 185, 186]. High IGF-II mRNA levels are associated with a more aggressive phenotype of ACC and a 5-fold increased risk of recurrence [180, 187] (Table 1).

In phosphoenolpyruvate carboxykinase (PEPCK) promoter human IGF-II transgenic mice, postnatal overexpression of IGF-II induced significantly increased adrenal weights, mainly caused by hyperplasia of the zona fasciculate [188]. This is in accordance with elevated serum corticosterone levels in IGF-II transgenic animals [189]. However, the observation that transgenic mice overexpressing IGFs or IGFBP-2 do not develop adrenal tumors indicates that IGF-II alone is not a tumor initiator for adrenal cells but rather a tumor progression factor that requires additional effectors for triggering adrenal tumorigenesis [189]. This notion is also supported by the clinical observation that deregulation of the IGF system is a late event often associated with advanced stage of the disease and poor clinical prognosis [118, 180, 190].

Indeed, in a cohort of pediatric and adult patients with adrenocortical tumors, IGF-II transcripts were mainly overexpressed in adult ACCs compared to adenomas [177]. Yet, a microarray analysis of 24 pediatric adrenocortical tumors (5 adenomas, 18 carcinomas, and 1 undetermined) demonstrated that the median expression of IGF-II in adrenocortical tumors was 18 times higher than in normal adrenal glands [191].

IGF-IR [192] and the IGF-binding protein-2 (IGFBP-2) [180] are also specifically overexpressed in ACCs. These molecular alterations may trigger a cascade of molecular events that can ultimately lead to malignancy in adrenocortical tumor progression [193]. This notion is confirmed by studies in adrenocortical tumor mouse cell line Y1, which have shown that stable transfection with human IGF-IR cDNA results in increased mitogenic response (+140%) to IGF-I as compared with nontransfected Y1 cells. In IGF-IR transfected cells the antiproliferative effect of ACTH was blunted and could be further antagonized by exogenous IGF-I [194] (Table 1).

In order to further clarify the significance of the IGF-IR in tumorigenesis of the human adrenal gland, Weber et al. examined the binding characteristics and concentrations of IGF-IR in normal adult human adrenocortical glands and in adrenocortical tumours of various origin. IGF IR binding in adrenocortical hyperplasias and adenomas was similar than in normal adrenocortical tissue. In contrast, three out of four hormonally active ACCs showed strongly elevated specific IGF-I binding with a 3-4-fold increase in IGF-IR concentration, as compared with normal adrenocortical tissue [194]. H295R cells overexpress also IGFBP-2 [195], which accounts for only 12% of the IGFBP activity in normal adrenocortical cells, but seems to play a specific role in the progression of ACCs by modulating IGF-II activity [196]. In support of the hypothesis of a tumor-growth-promoting effect of IGFBP-2 is the observation that Y-1 mouse adrenocortical tumor cells overexpressing IGFBP-2 show increased tumorigenic potential and cell proliferation [197]. However, the mechanisms of the IGFBP-2-associated increase in adrenal tumorigenesis remain largely unclear (see below).

Although the regulation of IGFBP production by IGFs is highly cell and species specific, a stimulatory effect of IGFs on IGFBP-3 has been reported in a large variety of cell systems [168, 198–204]. Treatment of adult human adrenocortical cells with ACTH predominantly stimulated the abundance of IGFBP-1 and to a lesser extent that of IGFBP-3, while IGF-I and IGF-II selectively induced the accumulation of IGFBP-3 and IGFBP-5 in the medium [205, 206]. Quantification of the specific bands by *γ* counting revealed that IGFBP-3 accounts for more than half of the detected IGFBP activity, followed by IGFBP-1 with 20% and IGFBP-4 with approximately 10% [196].

The gene expression profiles of IGFs system component may even distinguish malignant and benign tumours [207]. By analyzing the transcriptional profiles in 7 patients with ACCs and 13 with adenomas, Velázquez-Fernández et al. showed that in ACCs several IGF-related genes as IGF-II, IGF-IR, IGFBP3, and IGFBP6 were most significantly upregulated [207].

Recently, miRNAs able to regulate the IGF expression pattern in childhood adrenocortical tumors have been identified. Functional analysis of these miRNAs showed miR-99a and miR-100 regulate expression of IGF-IR, mTOR, and rictor in adrenocortical cancer cells, acting on target sites in their 3′-UTR regions. Downregulation of endogenous miR-100 in H295R and SW-13 cells increased protein expression of mTOR, raptor, and IGF-IR [208].

In order to evaluate the functional consequences of IGF-IR inhibition in adrenal carcinomas, Barlaskar et al. analyzed a large series of benign and malignant human adrenal tumors and a panel of ACC cell lines using a tyrosine kinase inhibitor, NVP-AEW541, and a fully human monoclonal antibody anti-IGF-IR, IMC-A12, both specifically targeting IGF-IR. Treatments with both NVP-AEW541 and IMC-A12 resulted in inhibition of growth of ACC cells *in vitro*. In xenograft tumors, IGF-IR blockade was more potent than mitotane, the first-line adrenolytic drug used in patients with ACC, and significantly enhanced mitotane response [209]. *In vitro* (in H295 and in SW-13 cells) efficacy of NVP-AEW541 treatment was confirmed by other studies [177, 210].

Thiazolidinediones (TZDs), a class of antidiabetic drugs, have also been investigated as potential therapeutic agents for ACC. TZDs are ligands for the peroxisome-proliferator-activated receptor (PPAR)-*γ*, a member of the nuclear receptor superfamily of ligand-dependent transcription factors, that is expressed predominantly in the adipose tissue but also in other tissues, although at much lower levels. PPAR-*γ*  exerts a critical role in several biological processes such as adipogenesis, glucose metabolism, inflammation, cell growth, and differentiation [211]. Nowadays, the molecular basis for the antitumor action of PPAR-*γ*  agonists remains incompletely elucidated. However, numerous studies support the notion that PPAR-*γ*  activation induces apoptosis and thus exerts anticancer effects [212]. Although no differences in the expression of PPAR-*γ*  are seen in normal and tumor tissue, the PPAR-*γ*  agonist rosiglitazone inhibited growth and invasiveness of H295R cells [213]. Indeed, both in SW-13 and H295 ACC cells, rosiglitazone inhibited the signaling pathways downstream IGF-IR, but not the receptor itself [179] (Table 2). 

Clinical trials are currently investigating the efficacy of monoclonal antibody IMC-A12, either used as monotherapy or in combination with mitotane (trials NCT00831844 and NCT00778817, resp.). The dual kinase inhibitor of both IGF-IR and IR, OSI-906, is currently being evaluated in ACC patients (trial NCT00924989) (Table 2).

### 4.3. Ovarian Cancer

Epithelial ovarian cancer (EOC) constitutes 90% of ovarian malignancies [214] and is the most common cause of gynecological cancer-related mortality [215]. It is fairly common in Scandinavia, less common in western Europe and North America, and infrequent in the developing countries and in Japan [216]. A first-degree family history of EOC is associated with approximately 3-fold increased risk [217]. EOCs are subdivided into four major categories: high-grade serous (70%), endometrioid (10%), clear cell (10%), mucinous (3%), and low-grade serous carcinomas (<5%) [214]. The marked clinical differences in ovarian cancer stage at presentation, response to therapy, and survival are manifestations of a complex underlying molecular heterogeneity of ovarian cancers [215]. Relatively little is known about the basic molecular and cellular mechanism that modulates growth of epithelial ovarian cancer and, presently, there are no available treatments capable of curing recurrent ovarian carcinomas due to their rapid evolution into a chemoresistant disease [218].

In physiology, as previously mentioned, regulation of ovarian activity requires a functional IGF axis. Moreover, over the last decade, accumulating data suggest that the insulin/IGF pathway might be a promising therapeutic target in ovarian cancer [219] (Table 1).

In 1991, Yee et al. examined the possibility that the IGF system could be important in regulating the autocrine growth of EOC cells [220]. The expressions of IGF-I-, IGF-IR-, and IGF-binding proteins were studied in ovarian cancer cell lines and tissues. IGF-IR mRNA was found in ovarian cancer cell lines and the primary or metastatic ovarian cancer tissues. In OVCAR-3 cell line, IGF-binding proteins, including IGFBP-2, IGFBP-3 and IGFBP-4, were expressed. In epithelial cells derived from untreated, ovarian cancer specimens, exogenous IGF-I induces cell proliferation. These cells secrete IGFs and IGF-binding proteins and express IGF-IR [221]. Ovary cancer cell lines also express IR; elevated levels of IR and insulin binding capacity were present in six cancer cell lines as compared to normal ovarian epithelium cell lines and were associated with mitogenic signaling in response to low doses of insulin. IR isoform analysis has shown preferential expression of IR-A, suggesting the ability of exogenous IGF-II to stimulate EOC cell proliferation through IR-A [54] (Table 1).

Moreover, studies in NIH-OVCAR3 cells have shown that IGF-I and IGFBP-2 promote ovarian cancer cell growth and invasiveness. IGFBP-2 is dramatically increased in the serum and ovarian cyst fluid of women with epithelial ovarian cancer [222, 223] and is involved in stimulation of cell growth [223]. In agreement with these data, elevated serum levels of IGF-I and IGFBP-2 have been associated with an increased risk of ovarian cancer [224, 225] (Table 1).

Other studies have shown that IGFBP-2 expression level in epithelial ovarian cancers is up to 38-fold higher than in normal ovarian epithelium [226]. Moreover, serum IGFBP-2 levels are elevated in women with early- and advanced-stage ovarian cancer as compared to controls and to patients with benign gynecological conditions, indicating that IGFBP-2 may be useful as a serum biomarker for detection and monitoring of epithelial ovarian cancer. Although the cellular mechanisms through which IGFBP-2 exerts a role in ovarian tumorigenesis are not completely elucidated, experimental data demonstrate that the growth-modulating effects of IGFBP-2 in ovarian cancer cells may be mediated by the activation of three specific cascades controlling cell growth, proliferation, and differentiation, that is, extracellular signal-regulated protein kinases (ERKs), stress-activated protein kinases (SAPKs) or c-Jun N-terminal protein kinases (JNKs) and p38 kinases. Furthermore, it has been seen that IGFBP-2 may regulate the expression of several potential cancer-promoting cytokines including fibroblast growth factors 6 and 7 (FGF-6 and -7), neurotrophin-4 (NT-4), and placental growth factor (PIGF) [223]. Thus, although IGFBPs are potent modulators of the mitogenic effects of IGFs, IGF-independent actions have also been recognized suggesting that IGFBPs are a separate class of growth modulators.

IGF-II is also considered a molecular marker and potential therapeutic target for the most aggressive EOCs. Indeed, when compared with normal ovarian surface epithelium samples, ovarian cancers show approximately 300-fold higher expression of the IGF-II gene. High IGF-II and lower IGFBP-3 expression are associated with high-grade, poorly differentiated, and advanced-stage disease [227, 228].

The association between IGF-II expression and ovarian cancer survival is driven by two specific promoters of IGF-II gene [229]. The IGF-II gene has four promoters, and each initiates a promoter-specific transcript which is expressed in a temporal and spatial-dependent manner. The transcription of three of the four IGF-II promoters, promoters 2, 3, and 4 (P2, P3 and P4), is regulated by DNA methylation [230]. DNA methylation alterations have been identified as being involved in tumorigenesis and disease progression [231, 232].

Using methylation-specific polymerase chain reaction (MSP) assay [233] it has been found that the methylation pattern of P2 and P3 IGF-II promoters as well as the levels of IGF-II mRNA and peptide was significantly different among patients with distinct tumor grade, residual tumor size, and treatment response. Patients with methylated P2 and unmethylated P3 (P2M/P3U) had 5 times higher mRNA expression and nearly 2-fold higher peptide levels compared to those with opposite pattern of methylation (P2U/P3M) [232].

Recently, genetic variations across the IGF components have been correlated with ovarian cancer risk [119, 120]. In primary ovarian cancer tissue, using microarray technology, Spentzos et al. have analyzed the expression patterns of gene families and pathways of IGF axis. Studying sixty-four patients with advanced stages of EOC, they found that expression patterns of IGF axis genes have prognostic significance in this highly lethal disease [234].

However, components of the IGF-I pathway were found overexpressed also in low-grade tumors, which respond to treatment with exogenous IGF-I with increased proliferation and migration [219].

Early data have shown that phosphorothioate antisense oligodeoxynucleotides (S-ODNs) [235] inhibit the function of the IGF-IR in NIH-OVCAR3 ovarian cancer cells and suppress cancer cell growth *in vitro*, but have small effects *in vivo* [235]. In the same cell model, a neutralizing antibody to IGFBP-2 also inhibits cell growth and downregulates the expression of a number of potential cancer-promoting cytokines [223].

More recently, it has been shown that NVP-AEW541, an IGF-IR tyrosine kinase inhibitor, is able to inhibit growth in EOC cell lines, OVCAR-3 and OVCAR-4, and to sensitize cells to cisplatin [236] (Table 2).

Today, at least five ongoing clinical trials aim to target the IGF-I axis in EOC patients. A phase II trial is currently investigating the fully human anti-IGF-IR monoclonal antibody AMG 479 in combination with paclitaxel and carboplatin (NCT00718523). Another phase II trial is examining AMG 479 in recurrent platinum-sensitive ovarian cancer (NCT00719212) (Table 2).

The combination between AMG 479 and AMG 655 (an human anti-DR5 monoclonal antibody) is the object of a study (phase I/II study) in patients with EOC and other advanced, refractory solid tumors (NCT00819169). A phase I/II trial is currently evaluating intermittent and continuous OSI-906, in combination with weekly paclitaxel, in patients with recurrent EOCs or other solid tumors (NCT00889382). Another phase I trial (NCT01322802) is testing the safety and immunogenicity of a DNA-plasmid-based vaccine encoding the amino acids 1–163 of IGFBP-2 in patients with advanced EOC.

Recently, an alternative therapeutical approach using insulin sensitizers, such as metformin, has been suggested against ovarian cancer. The rationale for using these drugs comes from evidence that a combination therapy with metformin and LY294002, an inhibitor of PI3K, reduces growth and induces apoptosis in ovarian cancer cells [237] by inhibiting PI3K/AKT and mTOR [238] while activating the AMPK/ACC pathway. In agreement with these preclinical data, an epidemiologic study conducted in 341 patients with EOC has shown that patients with T2DM who used metformin had longer progression-free survival than nonusers, despite receiving similar treatment for ovarian cancer [239]. A close relationship is now established between the use of metformin and progression, survival, and chemosensitivity of EOC [237–240] (Table 2).

Recently, a phase II clinical trial (NCT01579812) started to establish the potential role of metformin as anticancer stem cell agent in EOC patients. The primary objective of this study is to determine if metformin, administered as the time of traditional adjuvant chemotherapy to women with advanced EOC, will improve recurrence-free survival at 18 months compared to controls (Table 2).

## 5. Conclusions and Perspectives

Advanced endocrine tumors are characterized by poor prognosis and resistance to the common DNA-damaging chemotherapies or radiotherapy. Most extensively characterized endocrine malignancies include thyroid, adrenal, and ovarian cancers. In these tumors, a crosstalk between the IGF system and the pituitary hormones specific for each endocrine gland has been recognized and seems to exert a role in the tumorigenesis process. Recently, the risk of certain malignancies, including endocrine related cancers, has been found 2-3-fold increased in obese and T2DM patients. Insulin resistance and compensatory hyperinsulinemia, typical features of both obesity and diabetes, are the major candidates for cancer risk and are also associated with poor cancer prognosis and resistance to conventional and targeted anticancer therapies. Multiple alterations in the IGF system as well as association with high circulating levels of insulin/IGFs have been reported by several studies for these three endocrine cancer histotypes. This scenario may have important implications for endocrine cancer prevention and treatment. However, the potential role of the IGF system as therapeutical target in these tumors has being only recently evaluated and few clinical trials are currently ongoing.

Today the therapeutical strategies proposed to overcome IGF axis alterations in these malignancies include IGF-IR blocking antibodies, IGF-IR/IR tyrosine-kinase inhibitors, and insulin sensitizers. So far, preclinical results obtained with the first two classes of drugs mentioned above have shown promising hopes although the results are not conclusive and no complete responses have been reported. Furthermore, like in other malignancies, the development of intrinsic and adaptative resistance to IGF axis blockage could occur. Aberrant IR expression, particularly IR-A isoform, as well as HR-A formation and enhanced IGF-II autocrine production are very common alterations in endocrine cancers and could mediate the resistance to IGF-IR blocking drugs. Furthermore, insulin resistance and hyperinsulinemia are side effects of these drugs and may contribute to IR-A overactivation. New approaches aimed at specifically and safely targeting IR-A activation and/or disrupting the autocrine IGF-II/IR-A loop are urgently needed. In light of lines of evidence of association of endocrine cancers risk and hyperinsulinemia, insulin-sensitizers, such as metformin, hold promise as measures useful in cancer prevention.

## Figures and Tables

**Table 1:** Main molecular alterations involving the IGF system components in thyroid, adrenal, and ovarian cancer.

Molecular alteration	Thyroid cancer	Adrenal cancer	Ovarian cancer
IGF-IR overexpression	+	+	+
IR overexpression	+ (IR-A)	?	+ (IR-A)
HRs overexpression	+	?	?
IGFs overexpression	+ (IGF-I and IGF-II)	+ (IGF-II > IGF-I)	+ (IGF-II > IGF-I)
IGFs autocrine production	+ (IGF-I and IGF-II)	+ (IGF-II > IGF-I)	+ (IGF-I and IGF-II)
IGF-II/IR-A loop activation	+	?	+
IGFBPs overexpression	+	+ (IGFBP-2, IGFBP-3, IGFBP-6)	+ (IGFBP-2 > IGFBP-3 > IGFBP-4)
Relationship with elevated levels of insulin	+	+	+

**Table 2:** Preclinical and clinical studies.

	*In vitro*	*In vivo*	Trials	References
Thyroid cancer	Ab, IS	Ab, IS	—	Wang et al. 2006 [167]; Aiello et al. 2006 [169]; Chen et al. 2012 [170]
Adrenal cancer	TKI, Ab, IS	TKI, Ab	Ab, TKI	Barlaskar et al., 2009 [209]; Shen et al. 2007 [210]; Almeida et al., 2008 [177]; Ferruzzi et al., 2005 [213]; Cantini et al. 2008 [179]
Ovarian cancer	Ab, TKI, IS	IS	Ab, IS	Chakrabarty and Kondratick 2006 [223]; Gotlieb et al., 2006 [236]; Liao et al. 2012 [238]; Li et al., 2012 [237]; Romero et al., 2012 [239]

TKI: tyrosine kinase inhibitor targeting the IGF system; Ab: antibody; IS: insulin sensitizer.

## Abbreviations

RTKs: Receptors tyrosine kinase
SHC: Src homology domain C-terminal
Sos: Son of sevenless
Grb-2: Growth factor receptor-bound protein 2
PKA: Protein kinase A
PKC: Protein kinase C
G*α*s: G-protein (s) subunit alpha
Gaq: G-protein (q) subunit alpha
IRS-1: Insulin receptor substrate-1
PI3K: Phosphoinositide 3-kinase
PDK1: Phosphorylated 3-phosphoinoside-dependent protein kinase
Akt: Protein kinase B
mTOR: Mammalian target of rapamycin
Ras/Raf/MEK: Mitogen-activated protein kinase
ERK kinase: Extracellular-signal-regulated kinase
NF*κ*B: Nuclear factor kappa-B
p90RSK: p90 ribosomal S6 kinase
TFs: Transcription factors.
